# Transplantation of Bone Marrow-Derived Mesenchymal Stem Cells Promotes Delayed Wound Healing in Diabetic Rats

**DOI:** 10.1155/2013/647107

**Published:** 2013-04-09

**Authors:** Jiangbo Wan, Liulu Xia, Wenjia Liang, Yi Liu, Qian Cai

**Affiliations:** ^1^Department of Burns and Plastic Surgery, Lanzhou General Hospital of Lanzhou Command, 333 Binhe Road, Lanzhou, Gansu 730050, China; ^2^Department of Emergency, Baoji Central Hospital, Shaanxi 721008, China

## Abstract

In this paper, we established a delayed wound healing model on diabetic rat to mimic the pathophysiology of clinical patients who suffered from diabetic foot ulcers. We also evaluated if transplantation of allogeneic bone marrow-derived mesenchymal stem cells could promote the delayed wound healing and investigated the possible underlying biological mechanisms and stem cell behavior involved in this process. The results showed that bone marrow-derived mesenchymal stem cells had a positive effect on delayed wound healing in diabetic rats. Intramuscular transplantation demonstrated the best efficacy. This effect is associated with granulation tissue formation, angiogenesis, cellular proliferation, and high vascular endothelial growth factor expression in wound tissues. In addition, bone marrow-derived mesenchymal stem cells have been shown to mobilize and find home for ischemic and wounded tissues to participate in the process of wound healing. Intramuscular transplantation of exogenous isogeneic stem cells may be suitable for clinical application in the treatment of diabetic foot ulcers although the safety of this therapy should be considered.

## 1. Introduction

The incidence of diabetes mellitus is growing and reaching epidemic proportions worldwide [1]. The total number of diabetics is estimated to rise from 171 million in 2000 to 366 million in 2030 [2]. Diabetic foot ulcers (DFUs) are one of the most serious complications of diabetes. The lifetime risk of developing foot ulceration in persons with diabetes is as high as 25% [3]. Over 14–24% of these patients will have progressive disease that eventually leads to amputation [4]. In fact, complications of DFUs are the number one cause of nontraumatic lower extremity amputations [5], which is also associated with a high rate of morbidity and mortality with a 5-year survival rate as low as 31% for major limb amputees [6].

Wound healing is a complex process, which includes four overlapping phases: coagulation, inflammation, migration-proliferation, and remodeling [7]. Poor wound healing is a major issue in patients who suffer from DFUs. Peripheral vascular disease, trauma, infection, and neuropathy complicate the treatment of these wounds and thus necessitate a multidisciplinary approach [8]. Appropriate wound management varies mainly according to the cause of the wound, such as aggressive debridement, adequate pressure offloading, treatment of infection, hyperbaric oxygen therapy, bypass surgery for revascularization, and local dressings [9]. However, those concomitant or sequential therapeutic approaches are highly resistant and indolent in some cases, such as antimicrobial therapy aiming to cure the infection, not to heal the wound, while surgery to cure ulcers may result in secondary ulceration and other complications [10]. Therefore, there has been increased interest in novel therapies for DFUs that have been refractory to standard treatments.

Stem cell-based therapy represents a promising therapeutic approach for DFUs. Stem cells have been shown to mobilize and find home for ischemic and wounded tissues where they secrete chemokines and growth factors to promote angiogenesis and extracellular matrix remodeling [11, 12]. Several types of stem cells, such as BM-MSCs, have been reported to promote wound healing in DFUs [13–15]. These pluripotent stem cells are capable of differentiation into numerous cells types, including fibroblasts, osteoblasts, chondrocytes, adipocytes, myocardial cells, vascular endothelial cells, neurones, hepatocytes, epithelial cells, and other tissue cells [16, 17]. Many clinical trials also demonstrated that autologous BM-MSCs transplantation could improve wound healing in patients with DFUs [14, 18, 19]. However, the biological mechanisms for this improvement have not yet been identified.

In the present study, we established a delayed wound healing model in diabetic rats and evaluated the impact of allogeneic BM-MSCs transplantation on delayed wound healing and the possible underlying mechanisms of BM-MSCs in accelerating wound healing. We also determined which transplantation method is more effective in the improvement of wound healing.

## 2. Materials and Methods

This study was approved by the local animal ethics committee of Lanzhou General Hospital. All animals were treated humanely according to the guidelines for the care and use of laboratory animals published by the Chinese Ministry of Public Health.

### 2.1. Streptozotocin-Induced Diabetes

Diabetes was induced in four-month-old male Wistar rats of SPF grade (Experimental Animal Center of Traditional Chinese Medicine of Gansu Province, China). Briefly, rats were starved for at least 12 h before a single intraperitoneal injection of streptozotocin (STZ; Sigma, USA) dissolved in sodium citrate buffer (0.1 mM, PH 4.4) at a dose of 60 mg/kg body weight [20]. Seven days following STZ injection, blood samples were obtained from the angular vein, and the blood glucose levels were measured by glucometer. STZ-treated rats with blood glucose levels above 16.7 mmol/L were considered diabetic and were used in this study [20].

### 2.2. Establishment of a Delayed Wound Healing Model

The animal model was established on 36 diabetic rats and 12 age-matched nondiabetic rats by using previously described methods [21, 22]. Briefly, rats were anesthetized with an intraperitoneal injection of 10% chloral hydrate at 3 mL/kg body weight. The skin was disinfected with 75% ethanol, a rectangle was marked on the hind dorsal surface of the feet (both left and right) in each rat, and then a layer of skin in full thickness (standard area was 3 mm × 7 mm) was removed. The wound was then bandaged by sterilized dressing until BM-MSCs therapy was initiated. The day of BM-MSCs transplantation was defined as day 0. After surgery, the rats were returned to their cages in the animal holding room after they had regained consciousness and were housed separately.

### 2.3. Culturing of BM-MSCs

BM-MSCs were obtained by using previously described methods [23]. Briefly, 2 male Wistar rats (4-week-old) of SPF grade were sacrificed by C-spine luxation and soaked in 75% alcohol for 15 minutes. All femurs and tibias from both rats were taken and stripped of adherent muscles. A needle was inserted into the bone, and cells were aspirated followed by several flushes through the bone using a 1 mL syringe filled with culture medium, until all the bone marrow was flushed out of the bone. The marrow was scattered into single cell suspension by repeated flushes using 1 mL pipette filled with culture medium, and the residual marrow was filtered. The suspended cells were cultured by plastic adherence in Dulbecco's Modified Eagle Medium (Sigma, USA) with low glucose, 10% fetal bovine serum (FBS, Hangzhou Sijiqing Biological Engineering Materials Co., Ltd, China), and antibiotics. After 24 h culture at 37°C in a 5% CO_2_ atmosphere, the nonadherent cells were removed, and the media were changed every 3 days thereafter. When the cells were 80% confluent, adherent cells were harvested with 0.25% trypsin solution (Sigma, USA) for passage. Experiments in this study were carried out using cells from the third passage.

### 2.4. BM-MSCs Labeling

For *in vivo* tracing of BM-MSCs, the third-passage BM-MSCs were labeled with DAPI. Cells were incubated in the DAPI (20 *μ*g/mL [24]) containing medium for 2 h in the dark, following by thoroughly rinsing with PBS 5 times prior to trypsinization. Subsequently, DAPI-labeled allogeneic BM-MSCs (8.0 × 10^6^ cells/mL, according to our previous studies, data not shown) suspension was harvested for the following experiments.

### 2.5. Phenotypic Characterization of BM-MSCs

Before experimental use, the third-passage BM-MSCs were confirmed with their specific markers by flow cytometry. Briefly, a single cell suspension of 1.0 × 10^6^ cells/mL was placed in 100 *μ*L of PBS and was incubated in the dark for 30 min on ice with saturating concentrations of fluorochromes-conjugated mouse monoclonal antibodies against rat CD44-PE (Santa Cruz, CA, USA), CD90-PE, CD34PE, and CD45FITC (Becton Dickinson, CA, USA). And then cells were washed with PBS (1% FBS) 3 times, centrifuged at 1000 rpm for 5 min, and resuspended at a density of 1.0 × 10^6^ cells/mL. Cell fluorescence was evaluated immediately in a FACSCalibur flow cytometer (Becton Dickinson, CA, USA), and the data was analyzed using Cell Quest software (Becton Dickinson, CA, USA). Isotype-identical antibodies served as controls.

### 2.6. Experimental Design

Forty-eight male Wistar rats were randomly divided into four groups (*n* = 12 per group). In group I (non-diabetic controls) and group II (diabetic controls), rats were subcutaneously injected with PBS into their left hind legs and intramuscularly injected with PBS into their right hind legs. In group III, diabetic experimental rats were subcutaneously injected with DAPI-labeled BM-MSCs into their two hind legs. In group IV, diabetic experimental rats were intramuscularly injected with DAPI-labeled BM-MSCs into their two hind legs. On day 0, rats were treated with subcutaneous injections (6 sites, 5 mm away from the wound edge, 0.05 mL cells suspension or PBS per site) or by intramuscular injections (6 sites, 0.5 cm × 0.5 cm distance, 1 cm deep, 0.05 mL cells suspension or PBS per site). Three animals were sacrificed on each time point (days 2, 5, 8, and 11) after transplantation to evaluate the wound healing.

### 2.7. Estimation of Wound Contraction Rate

The size of wounded area was recorded by digital photographs taken from each rat's foot at each time point after injecting corresponding preparations (PBS for control group and BM-MSCs for experimental groups). Pictures were taken using a digital camera (DMC-LX5GK, Panasonic, Japan). The size of the wounded area was analyzed by Image-Pro Plus 4.5 software [25]. The rate of wound contraction was calculated by the following formula: Rate of wound contraction (%) = (original wound area − detected wound area)/original wound area × 100%.

### 2.8. Histological Examinations

At each time point, wound samples of each group were collected. These samples were divided into three parts. One part was cut in frozen sections, and the second part was fixed in 4% phosphate-buffered paraformaldehyde, embedded in paraffin, and sectioned for HE and immunohistochemical staining. The third part was preserved in liquid nitrogen for later ELISA and RT-PCR use. For histological examinations, the samples were sectioned with a thickness of 5 *μ*m. The frozen sections were immediately used to observe the trace of DAPI-labeled BM-MSCs and the intensity and distribution of blue fluorescence in wound tissues by a fluorescence microscope. The HE staining was used to detect granulation tissue formation.

### 2.9. Immunohistochemical Analysis

To study the angiogenesis during wound healing, the expression of CD31, a marker of endothelial cells, was detected using a polyclonal rabbit CD31 antibody (Santa Cruz, CA, USA). Cellular proliferation was assessed by the expression of Ki-67 using a monoclonal rabbit antibody (Abcam, UK). For the immunohistochemical staining, the paraffin sections (5 *μ*m) were deparaffinized, endogenous peroxidase was inactivated for 10 min with 3% H_2_O_2_, and the antigen was retrieved using sodium citrate buffer. After blocking with 5% BSA for 30 min, the slides were exposed to primary antibody at 37°C for 1 h. After washing with PBS, the slides were then incubated with biotinylated goat anti-rabbit secondary antibodies (Wuhan Boster Bioengineering Limited Company, China) at 37°C for 30 min, further developed with 3, 3′-diaminobenzidine tetrahydrochloride solution, and finally counterstained with hematoxylin. Positive staining was indicated by dark brown color. For immunostaining quantification, ten random digital images from each selected area were taken under 400x magnification. The number of CD31-positive small blood vessels and Ki-67-positive cells per high power field (HPF) were counted.

### 2.10. Measurement of VEGF Content in the Wound Tissues by ELISA

At every time point, wound tissue samples in each group were collected, and the level of VEGF in the wounded tissues was determined by using previously described methods [26]. Briefly, tissue samples were homogenized in 1 × PBS containing complete protease inhibitors (Wuhan Boster Bioengineering Limited Company, China). Homogenates were centrifuged (2500 rpm for 20 min) to remove debris, and the pellet was resuspended and filtered through a 1.2 *μ*m pore syringe filter. The level of VEGF was determined using a rat VEGF specific ELISA kit (Wuhan Boster Bioengineering Limited Company, China) according to manufacturer's protocol. The content of VEGF was expressed as pg/mg tissue.

### 2.11. Quantitative RT-PCR Analysis of VEGF mRNA Expression in Wound Tissues

Total RNAs of the wound tissues were extracted using RNAiso Plus (Takara, Japan) according to the manufacturer's instructions. 1 *μ*g of total RNA was used for reverse transcription into complementary DNA by PrimeScript RT reagent kit (Takara, Japan). Gene primers for rat VEGF and glyceraldehydes-3-phosphate dehydrogenase (GAPDH, internal control) were summarized in Table 1. Real-Time PCR was performed using SYBR Premix Ex Taq II (Takara, Japan). The samples were subject to the following conditions in 7300 Real-Time PCR System (Applied Biosystems, USA): after initial denaturation at 95°C for 30 s, PCR amplification was performed for 40 cycles at 95°C for 5 s and 60°C for 31 s. We selected the data of group II on day 2 as a calibrator. The equal efficiencies of amplification of the target and reference mRNA allowed for the comparative CT (2^−ΔΔCT^) method to be used to determine the relative level of gene expressions [27].

### 2.12. Statistical Analysis

Data were expressed as mean ± standard error of the mean (SEM). Statistical analysis was performed by one-way ANOVA when more than two groups were present and LSD-t for two groups (when there were differences between all groups). All statistical computations were performed using SPSS software (version 13.0). The significant level was set as *P* < 0.05.

## 3. Results

After the injection of STZ, an increase of blood glucose levels was observed over time, and this was accompanied by a reduction of body weight, polydipsia, polyuria, and increased diet. Blood glucose levels in diabetic rats used in the present study were consistently higher than 16.7 mmol/L, and these levels were not changed during the whole experimental process (data not shown). Diabetic rats did not gain weight after STZ injection and after BM-MSCs transplantation. Compared to their body weight pre-STZ injection, diabetic rats lost average 36 ± 5 g, 39 ± 7 g, 41 ± 10 g, and 43 ± 12 g body weight at 2, 5, 8, and 11 days after BM-MSCs transplantation, respectively, while the nondiabetic controls gained 20 ± 5 g, 22 ± 5 g, 26 ± 7 g, and 29 ± 8 g, respectively.

### 3.1. BM-MSCs Characteristics and DAPI Labeling

**Figure 1:**
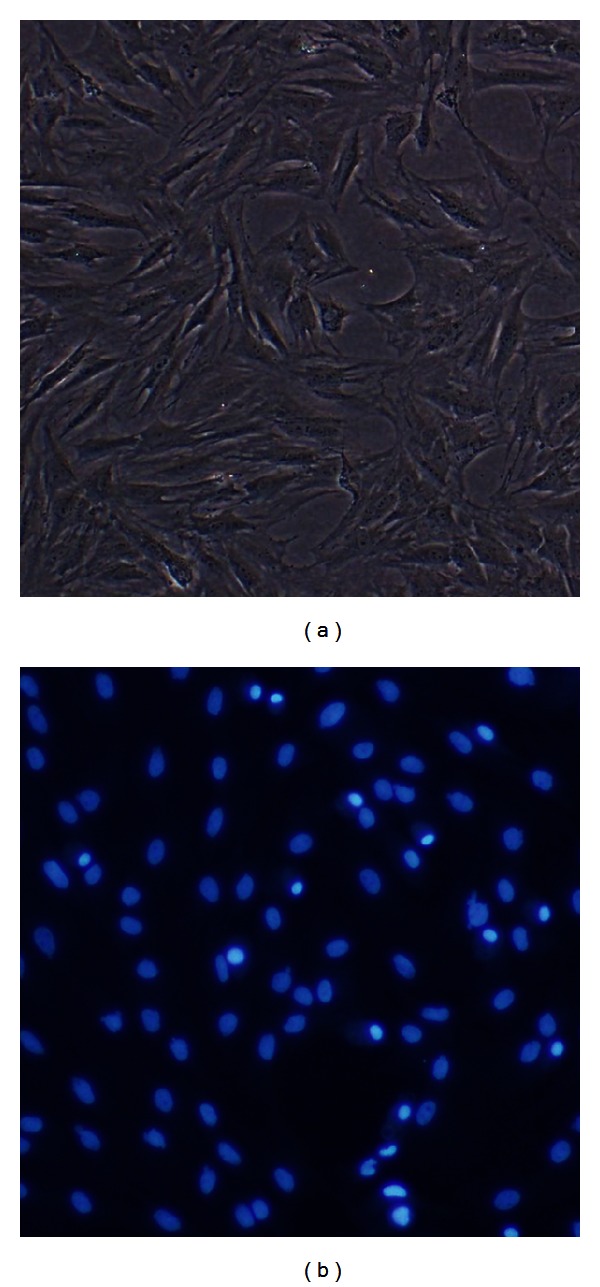
(a) The morphology of the third passage of BM-MSCs *in vitro*. (b) The third passage of BM-MSCs was incubated with 20 Ug/mL DAPI in culture medium for 2 h. Magnification: 100x.

After three passages in culture, the isolated cell population became homogeneous, showing a monolayer consisting of adherent cells displaying further traits of BM-MSCs, demonstrating a typical fibroblast-like morphology (Figure 1(a)). Adhesion to the bottom of the culture flask also served as a criterion to distinguish BM-MSCs from other free-floating cells. After being incubated in DAPI-containing medium for 2 h, the cell nuclei showed prominent blue fluorescence under ultraviolet light. We have also found that some cells have two micronuclei which were extremely bright and were probably just before cell divisions (Figure 1(b)).

### 3.2. Phenotypic Characterization of BM-MSCs

**Figure 2:**
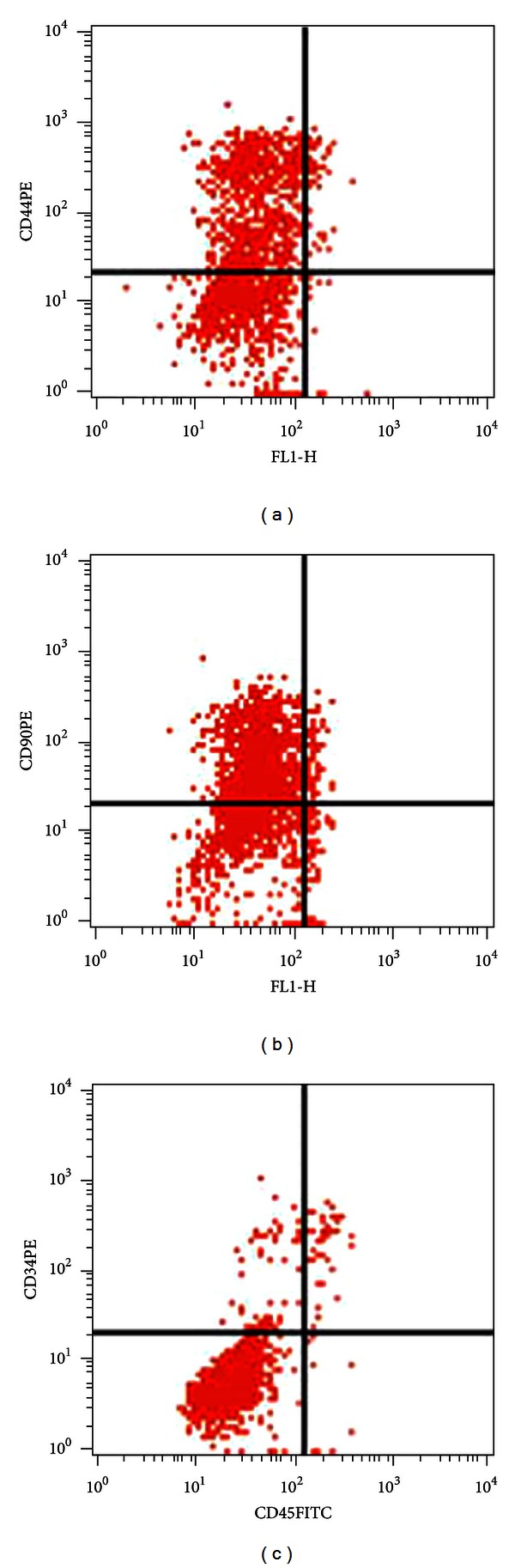
Flow cytometric analysis of BM-MSCs. Most of the cells were positive for markers CD44 and CD90 (72.07% and 89.53%, resp.) and were negative for markers CD34 and CD45 (1.55% and 2.16%, resp.).

Isolated BM-MSCs were confirmed by flow cytometry analysis using BM-MSCs specific markers. At passage 3, most of the BM-MSCs showed high levels of CD44 and CD90 expressions (72.07% and 89.53%, resp.) but were virtually negative for CD34 and CD45 expressions (1.55% and 2.16%, resp.), suggesting that the major population of the adherent cells were BM-MSCs (Figure 2).

### 3.3. BM-MSCs Enhance Wound Healing

**Figure 3:**
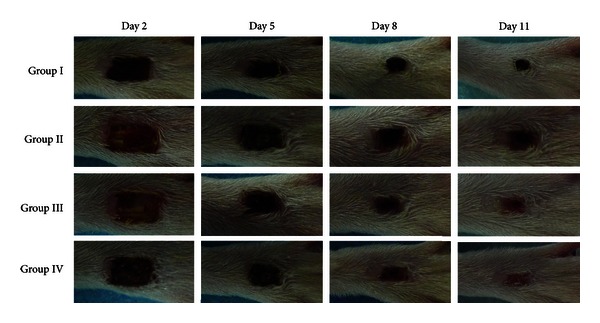
Representative images of wound healing at different time points after surgery. Apparently, the wound size was significantly reduced in non-diabetic rats (group I) as compared with other groups, while group IV was considered the second best group against groups II and III.

To compare the wound contraction rate in different groups, macroscopic gross differences of the wound surface were observed (Figure 3). On day 2, all the wounds were covered with a little purulent fluid. Redness and swelling were detected. On day 5, size of wound was significantly reduced with formation of black scabs and alleviation of the inflammatory responses in all groups. On days 8 and 11, some scabs become detached, and part of new epidermis appeared in groups I, III, and IV. But little hair was found on the new epidermis.  Table 2 summarizes the average rate of wound contractions in different groups. At every time point, the rate of wound contraction was higher in rats of group I as compared with other groups (*P* < 0.05). Furthermore, the wound area was smaller in group IV compared to that in groups II and III on day 11 (*P* < 0.05). However, we did not found statistical significance between groups II and III during the whole experimental process. These results suggested that intramuscular transplantation of BM-MSCs accelerated delayed wound healing in diabetic rats.

### 3.4. *In Vivo* Trace of DAPI-Labeled BM-MSCs

**Figure 4:**
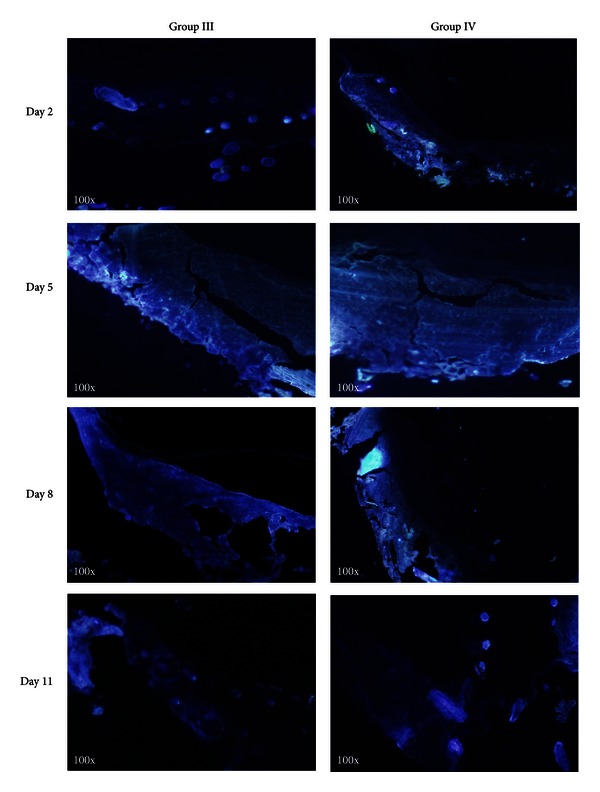
Frozen sections of groups III and IV showing the intensity and distribution of blue fluorescence (100x).

In order to trace the DAPI-labeled BM-MSCs in wound tissues, we made frozen sections on days 2, 5, 8, and 11, and the tissue was immediately observed under fluorescence microscope (Figure 4). There was no blue fluorescence at any time point in groups I and II (data not shown). While the intensity and distribution of blue fluorescence were obviously detected in groups III and IV, with the highest intensity observed on day 5, suggesting that the DAPI-labeled BM-MSCs migrated to the wound area to participate in the wound healing process, interestingly, the intensity and distribution of blue fluorescence in group IV were found much higher compared to that in group III on days 2 and 5.

### 3.5. BM-MSCs Improved the Thickness of Granulation Tissue

**Figure 5:**
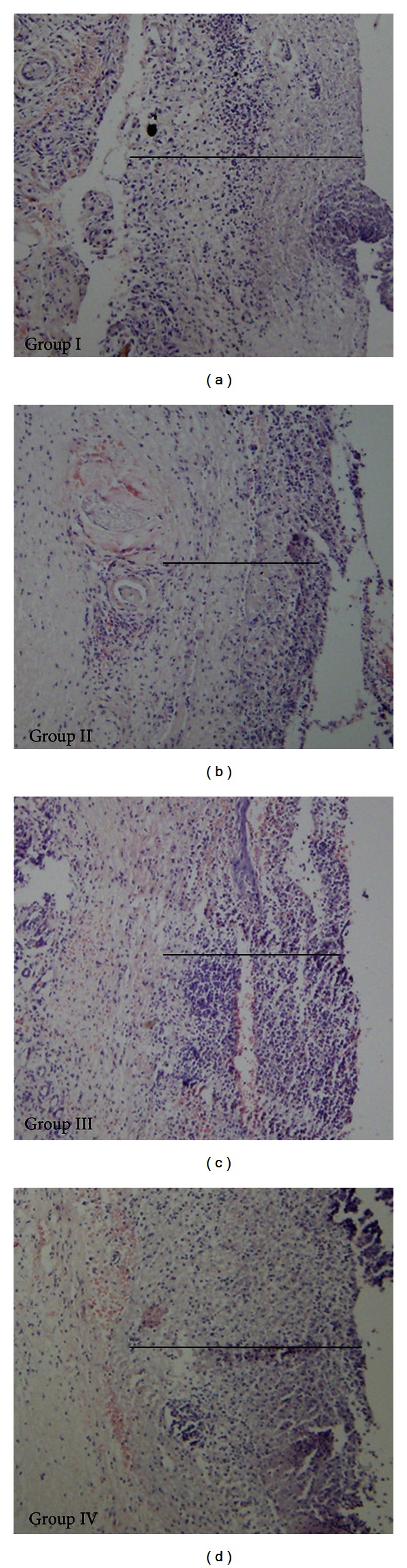
HE staining of sections showed that the granulation tissues in group I and group IV were thicker than that in groups II and III on day 5 (magnification: 100x). “-” represents the actual thickness of granulation tissue (292 ± 24 *μ*m, 221 ± 31 *μ*m, 272 ± 29 *μ*m, and 285 ± 27 *μ*m, resp.) in the pictures.

We also evaluated the impact of BM-MSCs on granulation tissue formation. As shown in Figure 5, the granulation tissue deposition showed no differences among these groups on day 2 (data not shown). However, the granulation tissue was thicker in groups I and IV compared to that in groups II and III on day 5. On days 8 and 11, reepithelialization around the wound edge was the predominant process during the late stage of wound healing. These findings suggested that more BM-MSCs may participate in and promote the formation of granulation tissue by intramuscular transplantation.

### 3.6. BM-MSCs Upregulate Angiogenesis and Cellular Proliferation

**Figure 6:**
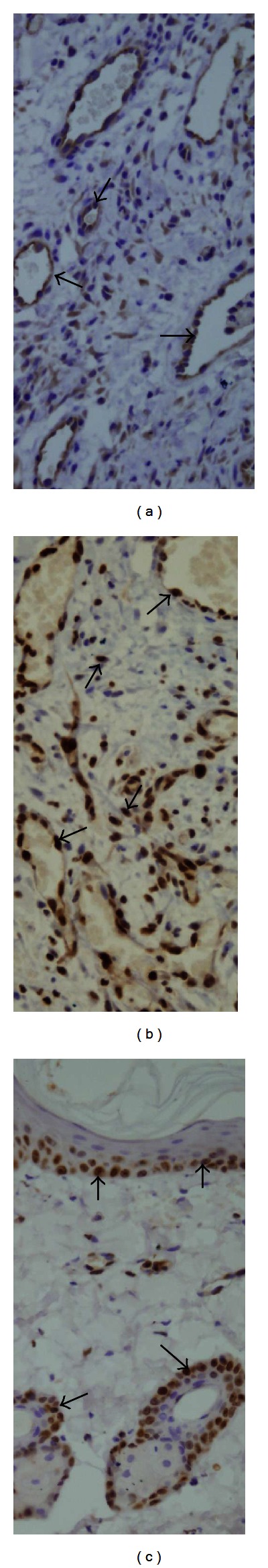
(a) Representative image of small blood vessels in group IV (400x). Black arrows indicate blood vessels. The positive staining of CD31 is on the membranes. ((b) and (c)) Two representative images of Ki-67-positive cells by immunohistochemistry in group IV (400x). (b) indicates the deep subcutaneous tissue which has Ki-67-positive endothelial vascular cells and fibroblasts. (c) indicates the basal epidermal layers and hair follicles which have Ki-67-positive basal cells and hair follicle epithelial cells. Black arrows indicate the Ki-67-positive nuclear staining.

Angiogenesis was analyzed in terms of CD31 expression levels in wounds. Small blood vessels of the wounds were characterized by CD31 immunohistochemical staining (Figure 6(a)). The average vessel density is quantified and summarized in Table 3. We found that the number of blood vessels increased along with the wound healing until day 8 in all groups. The angiogenesis in rats of group II (diabetic controls) was the weakest among those groups. On days 5 and 8, the vessel density in group IV was higher when compared with groups II and III (*P* < 0.05). And the increased number of small blood vessels in group III was detected compared to that in group II on days 8 and 11 (*P* < 0.05).

Next, we evaluated the cellular proliferation in the wound tissues using Ki67 staining (Figures 6(b) and 6(c)). The Ki67-positive cells were detected in vascular endothelial cells, fibroblasts, hair follicle epithelial cells, and basal cells. The average number of Ki67-positive cells in groups III and IV at every time point was significantly higher compared to group II (*P* < 0.01); no statistical significance was detected among group I, group III, and group IV (Table 4). Although those groups demonstrated angiogenesis and cellular proliferation in wound tissues and wound edge, intramuscular transplantation of BM-MSCs may result in the best efficacy of angiogenesis and cellular proliferation during wound healing.

### 3.7. BM-MSCs Improve the Contents of VEGF in Wound Tissues

To investigate the possible mechanism involved in the accelerated wound healing, we examined the expression level of angiogenic factor, VEGF in the wound tissues by ELISA.  Table 5 summarizes the wound tissue VEGF contents in these four groups. It is clear that the expression of VEGF augmented during wound healing process in groups I, III, and IV when compared with group II on days 5 and 8 (*P* < 0.01). On day 11, the VEGF level in group IV was significantly higher compared to that in the other three groups (*P* < 0.001).

### 3.8. Relative mRNA Expression Level of VEGF in Wound Tissues

To further confirm the results from ELISA, relative RT-PCR was employed to quantify the *in vivo* expression of VEGF mRNA. The results were summarized in Table 6. Relative values for VEGF expression were highest on day 8 in groups I, II, and III. However, the expression level of group II was always the lowest at all-time points. A clear trend of increase in VEGF expression was observed in group IV, and its expression level was significantly higher than that of other groups on day 11 (*P* < 0.001). These results confirmed that transplantation of BM-MSCs promoted the expression of VEGF in wound tissues, especially intramuscular transplantation of BM-MSCs at the later stage of wound healing.

## 4. Discussion

Normal wound healing is a dynamic and complex process involving a series of coordinated events, including bleeding and coagulation, acute inflammation, cell migration, proliferation, differentiation, angiogenesis, re-epithelialization, and synthesis and remodeling of the extracellular matrix [28]. Many factors can impair wound healing in DFUs, including infection, tissue hypoxia, necrosis, exudates, and excess levels of inflammatory cytokines, which prolong one or more phases of inflammation, proliferation, or remodeling [28]. The ideal modality of wound healing in DFUs should have synergistic effects of reducing inflammation, forming vital granulation tissue, and accelerating cellular proliferation and angiogenesis.

Functional characteristics of BM-MSCs that may benefit wound healing include their ability to migrate to the site of injury or inflammation, participate in regeneration of damaged tissues, stimulate proliferation and differentiation of resident progenitor cells, promote recovery of injured cells through growth factor secretion and matrix remodeling, and exert immunomodulatory and anti-inflammation effects [29–31]. In this study, transplantation of BM-MSCs demonstrated a positive effect towards enhancing delayed wound healing in diabetic rats. The major findings of this study are the following: (1) transplanted BM-MSCs highly migrated or found home for the wound tissues and contributed to tissue cells; (2) implantation of BM-MSCs promoted granulation tissue formation and re-epithelialization; (3) transplanted BM-MSCs significantly enhanced angiogenesis and cellular proliferation; and (4) a high expression of VEGF in wound tissue, especially in rats with intramuscular transplantation of BM-MSCs, was detected.

The wounds in our experimental rats demonstrated inflammatory characteristics, such as purulent fluid, redness, and swelling. Intramuscularly injected BM-MSCs can migrate from muscles to sites of injury in response to chemotactic signals to modulate inflammation and contribute to tissue remodeling. Many studies found that engrafted BM-MSCs can differentiate into keratinocytes, epithelial cells, and endothelial cells in the skin [12, 32, 33]. Differentiation and paracrine signaling have both been reported to implicate as mechanisms by which BM-MSCs improve tissue repair [34]. BM-MSCs differentiation contributes to regeneration of damaged tissues, whereas BM-MSCs paracrine signaling is likely the primary mechanism for reducing inflammation, promoting angiogenesis, and inducing migration and proliferation [35]. The primary method of wound healing in humans is granulation tissue formation and reepithelialization. Granulation tissue is essential to wound healing, as it is formed on the surface of wounds to protect and provide nutrition to wounds, consisting of fibroblasts, new capillaries, and infiltrated inflammatory cells. On day 5, the blue fluorescence was observed and peaked in granulation tissue and surrounding area on rats of groups III and IV. The granulation tissue was thicker than that in diabetic controls, indicating that transplanted BM-MSCs accelerated thicker granulation tissue formation and accelerated wound closure. 

One of the important components of granulation tissue is fibroblasts. Fibroblast senescence in chronic wounds is a contributory factor to poor healing [36]. Senescent fibroblasts produce elevated levels of the proteolytic enzymes, such as collagenase, elastase, stromelysin, and decreased levels of metalloproteinase inhibitors TIMP-1 and TIMP-3 [37]. In our study, cellular proliferation and regeneration were examined in terms of Ki-67 expression in wound tissues and wound margin area. Our results showed that not only fibroblasts but also endothelial vascular cells, basal cells, and hair follicle epithelial cells demonstrated increased proliferation during the healing process, suggesting that BM-MSCs transplantation promoted wound healing by increasing cell proliferation, which in turn facilitated the wound healing process and led to enhanced tissue regeneration. Another important component of granulation tissue is blood vessels which are necessary to support the formed granulation tissue. In this study, we demonstrated that BM-MSCs-treated wounds had enhanced capillary density, suggesting that BM-MSCs promote angiogenesis. Indeed, we also found increased VEGF levels in wounds of BM-MSCs transplanted rats compared to diabetic control rats. VEGF is one of the most important proangiogenesis factors that promote angiogenesis, stimulate cell migration and proliferation, delay senility, inhibit apoptosis, and promote cell survival [38–40]. Chronic wounds in diabetes are mainly due to the lack of angiogenesis [41]. Our results suggested that BM-MSCs transplantation in the wound accelerated release of VEGF in wound tissues, which may be partially responsible for BM-MSCs-mediated enhanced angiogenesis and wound healing.

Many scientists have employed different approaches to promote ulcer healing [42–45]. However, there is not yet an ideal therapy that is widely acceptable at present. Becaplermin is the only growth factor that has demonstrated therapeutic efficacy in randomized controlled trials of significant numbers of patients [46]. However, the cost of this treatment is high, and its half-time is short, which greatly limits its application. Stem cells are a particularly promising therapy for the treatment of chronic nonhealing wounds; the mechanisms should be revealed clearly to develop more efficient treatment strategies. Long-term systemic effects of stem cell therapy have yet to be established, and the security of stem cell therapy should also be of concern. More practical and promising therapies should be used in clinical treatment.

In conclusion, our study provides evidence that BM-MSCs are beneficial in the treatment of delayed wound healing in rats. And intramuscular transplantation of exogenous isogeneic stem cells may be suitable for clinical application in the treatment of DFUs, although the safety of this therapy should be considered. However, future studies are necessary to overcome the limitations of our experimental design, including clarification of the characteristics and the phenotype changes of these transplanted stem cells during the wound healing process and establishing a more suitable animal model for mimicking patients who suffered from DFUs.

## Figures and Tables

**Table 1:** Primers used for RT-PCR in this study.

Gene	Primers (F = forward; R = reverse)	Amplicon size (bp)
VEGF	F: 5′-GTCCTCACTTGGATCCCGACA-3′ R: 5′-CCTGGCAGGCAAACAGACTTC-3′	99
GAPDH	F: 5′-GGCACAGTCAAGGCTGAGAATG-3′ R: 5′-ATGGTGGTGAAGACGCCAGTA-3′	143

**Table 2:** Rate of wound contraction at different time points (*n* = 6, % of initial area).

Groups	Day 2	Day 5	Day 8	Day 11
I	18.70 ± 1.68*	53.12 ± 2.73*	79.01 ± 1.99*	91.27 ± 1.80*
II	12.85 ± 1.43	38.73 ± 1.89	64.44 ± 2.27	72.31 ± 2.82
III	13.38 ± 1.33	40.68 ± 2.09	69.11 ± 2.30	76.36 ± 2.04
IV	13.78 ± 1.20	45.45 ± 2.86	72.02 ± 1.80^&^	83.49 ± 2.01^#^

*P < 0.05, compared with groups II, III, and IV; ^&^ *P*< 0.05, compared with group II; ^#^ *P*< 0.05, compared with groups II and III.

**Table 3:** Number of the small blood vessels of wounds (*n* = 10, /HPF).

Groups	Day 2	Day 5	Day 8	Day 11
I	11.9 ± 0.7	23.6 ± 1.2^#^	25.5 ± 1.1^&^	18.3 ± 1.4^&^
II	10.3 ± 0.7	16.4 ± 0.8	20.2 ± 1.1	13.6 ± 1.1
III	10.8 ± 0.7	18.5 ± 1.1	23.7 ± 1.1^&^	18.2 ± 1.1^&^
IV	11.5 ± 0.6	21.9 ± 1.1^#^	27.7 ± 1.3^#^	19.8 ± 1.0^&^

^#^ *P* < 0.05, compared with groups II and III; ^&^ *P* < 0.05, compared with group II.

**Table 4:** Number of Ki-67-positive cells in wound tissue (*n* = 10, /HPF).

Groups	Day 2	Day 5	Day 8	Day 11
I	104.1 ± 4.5	114.8 ± 4.5^&^	119.6 ± 5.7^&^	97.0 ± 4.7
II	94.1 ± 4.7	98.8 ± 5.5	103.5 ± 6.1	85.5 ± 4.8
III	118.7 ± 5.6^@^	128.2 ± 5.9^@^	128.6 ± 6.4^@^	104.8 ± 5.5^@^
IV	114.4 ± 5.3^@^	123.1 ± 6.1^@^	126.3 ± 6.4^@^	106.3 ± 5.7^@^

^@^ *P* < 0.01, compared with group II. ^&^ *P* < 0.05, compared with group II.

**Table 5:** The content of VEGF (*n* = 6, pg/mg tissue).

Groups	Day 2	Day 5	Day 8	Day 11
I	0.44 ± 0.03^&^	0.85 ± 0.03^@^	1.02 ± 0.02^@%^	0.84 ± 0.02^&^
II	0.31 ± 0.02	0.47 ± 0.04	0.69 ± 0.03	0.66 ± 0.02
III	0.37 ± 0.03	0.77 ± 0.04^@^	0.90 ± 0.04^@^	0.81 ± 0.02^&^
IV	0.35 ± 0.04	0.79 ± 0.03^@^	1.03 ± 0.02^@%^	1.38 ± 0.02^$^

^&^ *P* < 0.05,   ^@^ *P* < 0.01, compared with group II; ^%^ *P* < 0.05, compared with group III; ^$^ *P* < 0.001, compared with groups I, II, and III.

**Table 6:** Relative mRNA expression levels of VEGF (*n* = 6, fold changes).

Groups	Day 2	Day 5	Day 8	Day 11
I	2.30 ± 0.04*	2.45 ± 0.06^@^	3.57 ± 0.12^@^	2.44 ± 0.06^@^
II	1.00 ± 0.05	1.41 ± 0.07	1.93 ± 0.10	1.31 ± 0.05
III	1.49 ± 0.09	2.23 ± 0.08^@^	3.31 ± 0.13^@^	2.27 ± 0.09^@^
IV	1.43 ± 0.08	2.31 ± 0.09^@^	3.99 ± 0.15^@%^	5.76 ± 0.22^$^

*P < 0.05, compared with groups II, III, and IV; ^@^ *P* < 0.01 compared with group II; ^%^ *P* < 0.05, compared with group III; ^$^ *P* < 0.001 compared with groups I, II, and III.
